# Labour analgesia: Recent advances

**DOI:** 10.4103/0019-5049.71033

**Published:** 2010

**Authors:** Sunil T Pandya

**Affiliations:** Department of Anaesthesia, Pain and Critical Care, Fernandez Hospital (Hospital for Women and Newborns) and Prerna Anaesthesia and Critical Care Services Pvt. Ltd., Hyderabad - 500 001, India

**Keywords:** Ambulatory epidurals, labour analgesia, recent advances

## Abstract

Advances in the field of labour analgesia have tread a long journey from the days of ether and chloroform in 1847 to the present day practice of comprehensive programme of labour pain management using evidence-based medicine. Newer advances include introduction of newer techniques like combined spinal epidurals, low-dose epidurals facilitating ambulation, pharmacological advances like introduction of remifentanil for patient-controlled intravenous analgesia, introduction of newer local anaesthetics and adjuvants like ropivacaine, levobupivacaine, sufentanil, clonidine and neostigmine, use of inhalational agents like sevoflourane for patient-controlled inhalational analgesia using special vaporizers, all have revolutionized the practice of pain management in labouring parturients. Technological advances like use of ultrasound to localize epidural space in difficult cases minimizes failed epidurals and introduction of novel drug delivery modalities like patient-controlled epidural analgesia (PCEA) pumps and computer-integrated drug delivery pumps have improved the overall maternal satisfaction rate and have enabled us to customize a suitable analgesic regimen for each parturient. Recent randomized controlled trials and Cochrane studies have concluded that the association of epidurals with increased caesarean section and long-term backache remains only a myth. Studies have also shown that the newer, low-dose regimes do not have a statistically significant impact on the duration of labour and breast feeding and also that these reduce the instrumental delivery rates thus improving maternal and foetal safety. Advances in medical technology like use of ultrasound for localizing epidural space have helped the clinicians to minimize the failure rates, and many novel drug delivery modalities like PCEA and computer-integrated PCEA have contributed to the overall maternal satisfaction and safety.

## INTRODUCTION

“The delivery of the infant into the arms of a conscious and pain-free mother is one of the most exciting and rewarding moments in medicine” Moir

Pain relief in labour has always been surrounded with myths and controversies. Hence, providing effective and safe analgesia during labour has remained an ongoing challenge. Historically, the era of obstetric anaesthesia began with James Young Simpson, when he administered ether to a woman with a deformed pelvis during childbirth. His concept of “etherization of labour” was strongly condemned by critics! The religious debate over the appropriateness of anaesthesia for labour[1] continued till 1853, when John Snow administered chloroform to Britain’s Queen Victoria during the birth of her eighth child, Prince Leopold.[2]

JY Simpson also proposed that “Medical men may oppose for a time the super-induction of anesthesia in parturition, but they will oppose it in vain; for certainly our patients themselves will force use of it upon the profession. The whole question is, even now, one merely of time.” This time came in the 1950s, when neuraxial techniques were introduced for pain relief in labour and, during the last two decades,[3] there have been several advances that lead to comprehensive and evidence-based management of labour pain.

Modern neuraxial labour analgesia reflects a shift in obstetrical anaesthesia, thinking away from a simple focus on pain relief towards a focus on the overall quality of analgesia.[4] The International Association for the Study of Pain (IASP) declared 2007–2008 as the ’’Global Year against Pain in Women - Real Women, Real Pain." The focus was to study both acute pain and chronic pain in women. Labour pain was found to be a good study model for treating acute pain. Increasing knowledge of the physiology and pharmacotherapy of pain and the development of obstetric anaesthesia as a subspecialty has improved the training in obstetric anaesthesia, leading to an overall improvement in the quality of labour pain relief.

In many countries today, the availability of regional analgesia for labour is considered a reflection of standard obstetric care. According to the 2001 survey, the epidural acceptance is up to 60% in the major maternity centres of the US. The National Health Services Maternity Statistics of 2005–2006 in the UK reported that one-third of the parturients chose epidural analgesia. In our country, the awareness is still lacking and, except few centres that run a comprehensive labour analgesia programme, the national awareness or acceptance of pain-relieving options for women in labour virtually does not exist.

## METHODS OF PAIN RELIEF IN LABOUR

### Nonpharmacological methods

Transcutaneous electrical nerve stimulation (TENS), continuous support in labour, touch and massage, water bath, intradermal sterile water injections, acupuncture and hypnosis, all may be beneficial for the management of pain during labour.[5] However, the number of women studied has been small and there have been no proven scientific data analysis of the quality of pain relief offered by these techniques. There is some evidence suggesting that water immersion during the first stage of labour reduces the use of epidural analgesia. A lack of data for some comparisons prevented robust conclusions.

### Parenteral narcotics

Systemic opioids have been used since 1840s and are the most widely used medications for labour analgesia.

Pethidine (meperidine), an opioid agonist, is the most frequently used opioid worldwide. Its effect on progress is contentious. Sosa *et al*.[6] have concluded that pethidine should not be administered in parturients with cervical dystocia as there is no benefit and that there is a greater risk of neonatal adverse outcome.

Intravenous ketamine, promoted by some clinicians as a sole anaesthetic for labour pains, is not safe as the labouring mother often requires anaesthetic dosages that may compromise the airway. Further, the benzodiazepines used to counteract delirium can cause neonatal respiratory depression. Its usage in labour should, therefore, be discouraged.

Fentanyl is a highly lipid-soluble synthetic opioid with analgesic potency 100-times that of morphine and 800-times that of pethidine.[7] Its rapid onset of action within 2–3 min after intravenous route with short duration of action and with no major metabolites makes it superior for labour analgesia. It can be administered in boluses of 25–50 µg every hour or as a continuous infusion of 0.25 µg/kg/h. Because of its pharmacokinetics and pharmacodynamics, it is suitable to be administered by patient-controlled intravenous analgesia (PCA).

Tramadol is a pethidine-like synthetic opioid having low affinity for mu(µ) receptors. Its potency is 10% that of morphine. It has no clinically significant respiratory depression at usual doses of 1–2 mg/kg body weight. The onset of action is within 10 min of intramuscular administration and the duration lasts for approximately 2–3 h. Claahsen-van der Grinten[8] demonstrated a high placental permeability for tramadol. However, neonates possess complete hepatic capacity to metabolize tramadol. Compared with pethidine, mothers receiving tramadol had higher pain scores. Therefore, crossover to alternate methods of relief is very common.

Butorphanol is an opioid with agonist–antagonist properties that resemble those of pentazocine. It offers analgesia with sedation. It is five-times as potent as morphine and 40-times as potent as pethidine. The dose of butorphanol is 2–4 mg intramuscularly. Butorphanol 2 mg produces respiratory depression similar to that with morphine 10 mg or pethidine 70 mg; however, there is a ceiling for respiratory depression at higher doses with butorphanol.[9] It is not frequently used for labour analgesia as it produces greater sedation.

### Remifentanil

Remifentanil is an ultra-short acting synthetic potent opioid. It has a rapid onset of action and is readily metabolized by plasma and tissue esterases to an inactive metabolite. The effective analgesia half-life is 6 min thus allowing effective analgesia for consecutive uterine contractions. It readily crosses the placenta, but is extensively metabolized by the foetus. Because of its pharmacokinetic profile, this agent has an advantage over other opioids for labour PCA.

The recommended dose of remifentanil is an intravenous bolus of 20 µg, with a lock out interval of 3 min on the PCA pump. In a study by Novelli *et al*.[10] on the efficacy and safety of intravenous infusion of remifentanil in 205 parturients, remifentanil was administered as a continuous infusion. The initial infusion of 0.025 µg/kg/min was increased in a stepwise manner to a maximum dose of 0.15 µg/kg/min. Maternal pain, other maternal and foetal variables, side-effects and satisfaction were recorded. The mean (±SD) visual analog score before the start of the infusion was 9.4 ± 1.2 cm, which decreased to 5.1 ± 0.4 cm after 5 min and 3.6 ± 1.5 cm after 30 min.

Most studies concluded that maternal monitoring during intravenous PCA with remifentanil should be one to one as maternal hypoventilation is more common and there are more episodes of oxygen saturation falling to <94% on pulse oximetry. However, it is a promising solution in women requesting labour analgesia, when neuraxial techniques are contraindicated.

### Opioid antagonists

Naloxone is the opioid antagonist of choice for reversing the neonatal effects of maternal opioid administration. It should be noted that there is no benefit of maternal administration of naloxone during labour or just before delivery. It is best to administer it directly to the new born if there is any neonatal respiratory depression. The dose of naloxone for reversing neonatal respiratory depression is 0.1 ml/kg. Administration of naloxone is not recommended during the primary steps of neonatal resuscitation. The preferred route of administration is the intravenous route. The intramuscular route is acceptable if intravenous access is not available, although the absorption is delayed. Endotracheal administration of naloxone is not recommended. Naloxone may precipitate a withdrawal in the new born of the opioid-dependent mother.[11]

For reversing maternal respiratory depression, the dose is 0.4 mg intravenously. It should be noted that it also reverses the analgesic action. The half-life of naloxone is shorter and repeat administrations may be required if the duration of action of the narcotic is longer.

## INHALATION METHODS

The only agent that has survived the test of time is nitrous oxide (Entonox), which is administered as 50:50 mixtures of oxygen and nitrous oxide. Other agents that have been tried in the recent years are the volatile anaesthetic agents sevoflurane (Sevox), isoflurane and enflurane.

### Entonox

A systemic review of the use of entonox in labour[12] concluded that entonox is certainly not a potent analgesic. Studies suggest beneficial effects on parturients if the method of inhalation is properly followed. Places where neuraxial techniques are not practiced, and in parturients with short labour, entonox inhalation is a useful method. The Obstetric Anaesthesia Association, UK (2005) guidelines state that entonox is being phased out from the UK in view of the poor analgesic efficacy and environmental pollution.

### Sevox – Patient-controlled inhalation analgesia

Sevoflurane is a volatile inhalational agent commonly used during general anaesthesia. Because of its short onset and offset of action, it appears to be the best-suited inhalational agent for labour analgesia and can be administered as patient-controlled inhalation analgesia.[13] It is used in the concentration of 0.8% with oxygen and needs specialized equipment. Further, there is a concern for environmental pollution and maternal amnesia and loss of protective airway reflexes. Larger studies are needed to assess the incidence of maternal compromise.

## REGIONAL ANALGESIA IN LABOUR

Central neuraxial analgesia is the most versatile method of labour analgesia and the gold standard technique for pain control in obstetrics that is currently available.[14] The use of neuraxial techniques has increased dramatically in the last 20 years, especially in the west, and few dedicated centres in India. It is unlikely that this will change soon as compared with other techniques. The satisfaction of birth experience is greater with neuraxial techniques.

There have been several exciting advances in the field of neuraxial analgesia[15,16] in terms of refinement of techniques [sequential combined spinal epidural analgesia CSEA)] and availability of newer drugs and adjuvants. The technological advances have facilitated the various modalities of novel drug delivery systems, like patient-controlled infusion regimes, and newer randomized controlled trials (RCTs) have helped to solve several controversies associated with neuraxial analgesia. The recent advances in neuraxial analgesia are tabulated in Table 1.

**Table 1:** Recent advances in neuraxial analgesia

Technical advances
Combined spinal epidural analgesia
Continuous spinal analgesia using microcatheters
Ambulatory epidurals, concept of MLAV and MLAD, low-dose and ultra-low-dose epidurals
Pharmacological advances
Ropivacaine, levobupivacaine
Newer opioids: sufentanil, remifentanyl
Adjuvants: clonidine and neostigmine
Technological advances
Availability of ultrasound to facilitate localization of epidural space, minimizing failures
Patient-controlled epidural analgesia regimes
Newer insights into the myths and controversies associated with neuraxial techniques
Effect and timing of epidural on caesarean section, maternal and neonatal outcome, breast feeding
Witholding the dose in the second stage of labour
Intrathecal placement of epidural catheter for reducing the incidence of PDPH in the event of inadvertent dural puncture
Role of CT scans and MRI in detecting complications associated with neuraxial blocks

MLAV, minimal local anaesthetic volume; MLAD, minimal local anaesthetic dose

## TECHNICAL ADVANCES

### CSEA technique and low-dose epidural regimes

With the evolution of sequential “needle-through-needle” combined spinal epidural technique, it can be safely used to provide labour analgesia. It combines the rapid, reliable onset of profound analgesia resulting from spinal injection with the flexibility and longer duration of epidural techniques.[16]

The CSEA kit spinal needle is a fine pencil-point needle that comes with a locking device, which minimizes postdural puncture headache and failed spinals. Use of the spinal opioids provides immediate analgesia without producing any motor block thus producing an ambulatory block. The epidural catheter is activated with low-dose mixtures of opioid and local anaesthetics; hence, the ability to walk is not impaired.

A review of the complications has concluded that CSEA is as safe a technique as a conventional epidural technique and is associated with greater patient satisfaction. There were no differences in maternal satisfaction, mode of delivery and ability to ambulate between CSEA and epidural techniques.[17,18] Side-effects and complications, however, can occur, which include pruritus, nausea and vomiting, hypotension, uterine hyperstimulation and foetal bradycardia and maternal respiratory depression. Foetal bradycardia is more pronounced with intrathecal sufentanil, perhaps due to its associated decrease in maternal catecholamines, which may precipitate uterine hypertonicity and foetal bradycardia.[19,20] However, several recent reports have found neither an increase in these complications nor an increase in the caesarean section rate.

The Obstetric Anaesthetists Association, UK guidelines in 2005[21] restrict the use of CSEA as a routine and are indicated only in certain specific situations, like very early stage of labour where local anaesthetics are avoided, advanced stages of labour where rapid analgesia is desirable and difficult epidurals as CSEA reduces the failure rate of epidurals.

### Low-dose epidural regimes

With the emerging concept of low-dose and minimal local anaesthetic dose and volumes (MLAD and MLAV), all present-day labour epidurals are low-dose epidurals. Traditionally, a high concentration (0.2–0.25%) of local anaesthetic has been used to maintain labour epidural analgesia. In the last decade, the concentration of local anaesthetic used to maintain labour epidural analgesia has been decreasing (0.0625–0.125%). The use of a low concentration of local anaesthetic has reduced the total dose of local anaesthetic used as well as the side-effects, such as motor blockade.[22,23]

Using an up–down sequential allocation method, Gordon Lyons *et al*.[24] in their comparative study sought to determine the minimum local analgesic volume (MLAV) and minimum local analgesic dose (MLAD) of an initial bolus of epidural bupivacaine 0.125% and 0.25%. The MLAV of bupivacaine 0.125% was 13.6 ml (95% CI 12.4–14.8) versus 9.2 ml (95% CI 6.9–11.5) for bupivacaine 0.25% (*P* = 0.002). Hence, by reducing the concentration, an equivalent labor analgesia was achieved with a significant reduction in the dose of bupivacaine. Such reductions in dose without compromising the analgesic efficacy provide a greater margin of safety and allow fine-tuning of labor analgesia. A RCT on combined obstetric mobile epidural trial (COMET) published in Lancet in 2001 by the UK Study Group concluded that low-dose epidural analgesia resulted in significantly higher vaginal delivery.[25,26]

### Maintenance of intrapartum neuraxial analgesia

Maintenance of intrapartum analgesia is either performed by intermittent manual boluses or through patient-controlled or continuous epidural infusion pumps. Several studies addressed the technical aspects of neuraxial anesthetic delivery systems.[27]

### Continuous epidural infusion of dilute local anaesthetic with opioid

Continuous dilute low-dose mixtures have been a major advance during the last few years. The dosage recommended for labour analgesia is 0.0625% bupivacaine with 2 mcg/ml of fentanyl, infusing at 10–12 ml/h.[28] Maternal and neonatal drug concentrations have been tested and have been demonstrated to be safe for both the mother and the neonate. These infusions have provided better pain relief but at the cost of more numbness and motor blockade and more breakthrough top-ups. Thus, the total dosage of local anaesthetic is higher when compared to patient-controlled epidural analgesia (PCEA) or intermittent bolus methods.

## PCEA

PCEA is a novel method of the drug delivery system, providing several advantages, including the ability to reduce the drug dosage. Self-control and self-esteem may be vital for a positive experience in childbirth, and PCEA achieves both. Thus, it is a useful alternative for the maintenance regime.

The ideal PCEA regimen is controversial. Lim *et al*.[29] in their study demonstrated that demand-only PCEA (5-ml bolus, 15-min lockout interval) resulted in less local anaesthetic consumption but an increased incidence of breakthrough pain, higher pain scores, shorter duration of effective analgesia and lower maternal satisfaction when compared with PCEA with background infusion (5-ml bolus, 10–12-min lockout interval and 5–10 ml/h infusion)

### Computer-integrated PCEA

Lim *et al*.[30] reported another adaptation of the epidural delivery pump technology. Their centre has developed a computer-integrated PCEA (CI-PCEA) that controls background infusion rates depending on the previous hour’s demand boluses. This randomized trial compared a standard PCEA technique of 0.1% ropivacaine with fentanyl administered as bolus-only by patient demand to the CI-PCEA technique that initiated an infusion algorithm with changing infusion rates depending on the demand boluses. Despite patients with the CI-PCEA technique receiving background infusions, the hourly consumption of ropivacaine was no different from that of the standard group. These studies illustrate that there is room for improvement in administering epidural medication, especially for women with prolonged labours.

### Continuous spinal analgesia with microcatheters

The Food and Drug Administration (FDA) has restricted the use of spinal microcatheters due to an association with the cauda equina syndrome. The FDA authorized a large randomized, double-masked,[31] multicenteric study to evaluate the safety of continuous intrathecal labour analgesia using a 28-gauge catheter versus continuous epidural labour analgesia. The results of the trial were able to rule out the association of this technique with neurologic injury. The study concluded that providing intrathecal labour analgesia with sufentanil and bupivacaine via a 28-gauge catheter has an incidence of neurologic complication <1% and that it produces better initial pain relief and higher maternal satisfaction, but is associated with more technical difficulties and catheter failures compared with epidural analgesia. Also, the CSEA kit is more expensive and hence is not routinely recommended.

There have been few studies using single-shot spinal analgesia using intrathecal morphine (300–400 mcg) with local anaesthetic. The analgesia is not satisfactory during the advanced stage of labour and the incidence of nausea and pruritus are unacceptably high. Hence, this is not routinely recommended.

## NEWER LOCAL ANAESTHETICS AND ADJUVANTS–CLONIDINE AND NEOSTIGMINE

The availability of newer local anaesthetics like ropivacaine and levo bupivacaine have contributed towards the increased maternal safety in terms of being less cardiotoxic after an inadvertent intravenous injection. However, for the dosage used for labour analgesia, cardiotoxicity is not a major issue. The recent study comparing these two drugs with bupivacaine offers no added advantage and is five-times more expensive as compared with that of bupivacaine. Atie´nzar and Parlance[32] in their randomized study comparing levo bupivacaine, ropivacaine and bupivacaine with fentanyl for labour analgesia concluded that all three regimens were effective during the first stage of labour, although pain scores were higher in those receiving levo bupivacaine. Motor block was greater with bupivacaine than with levo bupivacaine.

α-2 agonist, clonidine and cholinesterase inhibitor, and neostigmine have been used as adjuvants for labour analgesia.[33,34] Both the drugs possess a common mechanism of action that can be beneficial. They can be administered either epidurally or via the intrathecal route. Spinal clonidine, in doses of 100–200 mcg, produces excellent labour analgesia of short duration but at the cost of more sedation and hypotension. Spinal clonidine in doses of 50 mcg administered with bupivacaine and sufentanil mixture significantly prolonged the labour analgesia (197 compared to 137 min) without producing serious adverse effects. It has also been used with ropivacaine–fentanyl and was found to prolong the duration. However, the need of ephedrine requirement was higher in the clonidine group. Also, the foetal heart rate abnormalities were higher in the clonidine group.

Dewandre *et al*. in their study,[35] concluded that hypotension occurs more frequently when clonidine is added to epidural ropivacaine instead of an equi analgesic dose of sufentanil. Therefore, clonidine cannot be recommended for routine administration for labour epidural analgesia. Clonidine is not approved for use in obstetric patients in the United States.

## USE OF ULTRASOUND TO STUDY NEURAXIAL ANATOMY AND LOCALIZE EPIDURAL SPACE

Ultrasound imaging of the spine has recently been proposed to facilitate identification of the epidural space and predict difficult spine score, especially in women with abnormal lumbosacral anatomy (scoliosis) and those who are obese. Carvalho *et al*.,[36] in their study, found a good level of success in the ultrasound-determined insertion point and very good agreement between ultrasound depth (UD) and needle depth (ND). They also concluded that the proposed ultrasound single-screen method, using the transverse approach, can be a reliable guide to facilitate labour epidural insertion. Thus, the epidural failure rate can be minimized in patients with difficult backs.

## NEWER INSIGHTS INTO THE MYTHS AND CONTROVERSIES

### Increased rate of operative and instrumental delivery: Is epidural the cause?

The Cochrane Database Systemic trials have emphasized that epidural analgesia had no statistically significant impact on the risk of caesarean section. In two different metaanalyses of randomized trials, comparing patients with and without epidural, caesarean delivery was clearly not associated with epidural analgesia, which showed that there is no direct relationship of epidural and increased caesarean section.[37]

Use of neuraxial analgesia, however, is known to prolong the duration of labour on an average by 1 h. The association of occipito posterior position, augmentation with oxytocin and instrumental delivery is relatively higher in patients receiving epidural analgesia. However, the use of low-dose mixtures has reduced the overall incidence of these undesirable adverse effects. In a large randomized trial involving 1,054 patients (COMET study), the introduction of a low dose of epidural infusion was associated with a 25% decrease in the instrumental vaginal delivery.[25]

### Timing of epidural during labour: Epidural taken early vs. late

Most observational studies show a higher rate of caesarean delivery when epidural is initiated early in labour. The ACOG (ACOG Statement in 2000 - Evaluation of Caesarean Delivery) had suggested that epidural analgesia may be delayed until a cervical dilation of 4–5 cm is reached based on a study published by Thorp *et al*.[38] and few other studies.

However, the small degree of difference in cervical dilation between early and late groups (approximately 1 cm) is an important limitation of these trials. Wong *et al*.,[39] in their landmark RCT of nearly 750 primigravid women in early labour, concluded that there was no difference in the operative delivery of caesarean rates when neuraxial analgesia was administered early in labour (2 cm) vs. a group where epidural analgesia was administered late in labour (4–5 cm). Another study that used conventional epidural also had similar conclusions.

After the above evidence and several other metaanalyzed studies, the ACOG committee revised their statement, no longer endorsing a delay and explicitly disavowing the consideration of fear of increasing the risk of caesarean delivery. The ACOG and the American Society of Anesthesiologists (ASA) have also jointly emphasized that there is no need to wait arbitrarily till the cervical dilation has reached 4–5 cm, and endorsed a statement that “*Maternal request is a sufficient indication for pain relief in labour.*”[40]

### Early vs. delayed pushing

Delayed pushing has been advocated in parturients under neuraxial blockade. Passive descent should be encouraged along with delayed and monitored pushing during birth to safely and effectively increase spontaneous vaginal births, decrease instrument-assisted deliveries and shorten the pushing time.[41] The Pushing Early or Pushing Late with Epidural (PEOPLE) Study also supported delayed pushing for a better outcome.[42]

### Withholding the epidural top-up in the second stage

Many centres discontinue epidural analgesia late in labour to improve a woman’s ability to push and reduce the rate of instrumental delivery. But, in the RCTs of epidurals discontinued late in labour compared with continuation of the same epidural protocol until birth (462 participants), the reduction in the instrumental delivery rate was not statistically significant.[43] However, there was a statistically significant increase in inadequate pain relief when the epidural was stopped (22% vs. 6%, RR 3.68, 95% CI 1.99–6.80).

### Vaginal birth after caesarean and epidural

Task force guidelines 2007 jointly issued by the ASA and the Society of Obstetric Anaesthesiologists and Perinatologists (SOAP) recommend neuraxial techniques being offered to patients attempting vaginal birth after previous caesarean delivery. For these patients, it is also appropriate to consider early placement of a neuraxial catheter that can be used later for labour analgesia or for anaesthesia in the event of operative delivery.

### Epidural and breastfeeding

The effect of epidural analgesia on breastfeeding continues to appear in the lay press, in part due to conflicting reports in the scientific literature. Several studies and trials failed to demonstrate a significant association between epidural and lactation failure or less-successful breastfeeding attempts.[44] Further studies are needed in this area to assess the strength and the impact of any association, if any.

### Backache and epidural

In two recent randomized trials, there were no significant differences in the incidence of long-term back pain between women who received epidural pain relief and women who received other forms of pain relief.[45]

### Maternal pyrexia and the newborn

Epidural analgesia in nonobstetrical patients is generally associated with a slight decrease in body temperature secondary to peripheral vasodilation and redistribution of heat from the core to the periphery. In contrast, observational and randomized studies in obstetric patients demonstrate that epidural analgesia during labour is associated with maternal pyrexia and increased neonatal sepsis workup. The exact cause of maternal pyrexia is not known. The temperature rise generally is never above 1°C with epidural, sometimes observed in women with long labours. Always rule out and treat any underlying cause if the temperature rise is more than 1°C. Irrespective of the cause, any pyrexia during the intrapartum period needs to be aggressively treated with hydration, antipyretics and other appropriate measures. Intrapartum pyrexia due to epidural does not warrant evaluation for neonatal sepsis.[44] Further studies are needed to determine the criteria for performing workups for sepsis in infants of low-risk women who deliver infants at term.

### Postdural puncture headache

The use of small-bore “atraumatic” spinal needles will reduce the incidence of postdural puncture headache (PDPH) in patients receiving CSEA to approximately 1% or less. It was suggested that the incidence of unintentional dural puncture is less in CSEA patients than in patients receiving conventional epidurals as the spinal needle may be used for verification of correct placement of the epidural needle. Intrathecal placement of the conventional epidural catheter in case of inadvertent dural puncture reduced the incidence of PDPH.

### Advances in the management and treatment of complications following neuraxial blocks

Lipid rescue for treating local anaesthetic toxicity after an inadvertent intravenous injection was a new discovery. Weinberg *et al*. in their animal study model published in Anaesthesiology in 2008 concluded that all the metrics of resuscitation were much better with lipid infusion, which chelates bupivacaine from systemic circulation effectively and stabilizes haemodynamics better compared with epinephrine.

Newer mechanisms of nerve injuries were studied by Claudio *et al*. Pressures generated during the epidural injection were found to be in the range of 15–20 psi and in 10% of the patients, they were found to be around 30 psi. Eleven pounds per square inch are the break point for permanent nerve injury in case of intraneural nerve injections. Hence, this was a revelation that no injection or top-ups should be given when the patient experiences pain or paraesthesia during injection, a very important risk management strategy.

The infective complications (meningitis) following CSEA, which were reported to be high during the initial observational studies, still remains inconclusive. Presently, it is stated that infective complications are no more than what is being reported with other neuraxial techniques.

Insertion of epidural catheter more than 5 cm inside the epidural space can cause knotting and looping of catheters in the epidural space. Fluoroscopy or radiograph may not be helpful in locating the catheter. Computed tomography (CT) helps in locating the knotted or torn epidural catheter. The literature reports a case of catheter knot in the epidural space as well as a loop within the interlaminar ligamentum flavum between L3 and L4, visualized by CT.[46]

### Pharmacogenetics

Pharmacogenetics, or the study of how genes affect the response to drugs, offers the potential to tailor medications to each individual’s genetic profile. A significant increase in sensitivity to the analgesic effect of intrathecal fentanyl in labouring women carrying a common variant of the *µ*-opioid receptor gene was shown.[47] This demonstration of a 1.5- to 2-fold difference in analgesic requirement according to genotype is clinically relevant, because the provision of optimal labour analgesia remains a challenge, with a need to reduce doses and minimize opioid-related side-effects.

## CONCLUSION

The most important contribution of recent obstetric anaesthesia research to clinical practice has been the demonstration that early neuraxial labour analgesia does not negatively affect the mode of delivery and, obviously, improves maternal satisfaction. Other immediate applications relate to the choice of rather larger doses of more dilute solutions of bupivacaine–opioid mixtures for initiation and maintenance of labour analgesia using PCEA. The next generation of pumps might allow the automated delivery of “mandatory” boluses rather than background infusions to ensure a better spread of the infusate and, perhaps, utilize algorithm-based CI-PCEA programs.

The use of ultrasound guidance and continuous intrathecal analgesia via microcatheters offer the potential to overcome difficulties in neuraxial analgesia/anaesthesia placement in difficult cases.
